# *In Vitro*/*Vivo* Activity of Potential MCR-1 Inhibitor in Combination With Colistin Againsts *mcr*-1-Positive *Klebsiella pneumonia*

**DOI:** 10.3389/fmicb.2018.01615

**Published:** 2018-07-17

**Authors:** Yonglin Zhou, Tingting Wang, Yan Guo, Shui Liu, Jianfeng Wang, Yingbo Shen, Shusheng Tang, Yang Wang, Xuming Deng

**Affiliations:** ^1^Department of Respiratory Medicine, The First Hospital of Jilin University, Changchun, China; ^2^Key Laboratory of Zoonosis, Ministry of Education, Institute of Zoonosis, College of Veterinary Medicine, Jilin University, Changchun, China; ^3^Beijing Key Laboratory of Detection Technology for Animal-Derived Food Safety, College of Veterinary Medicine, China Agricultural University, Beijing, China

**Keywords:** *K. pneumonia*, MCR-1 inhibitor, pterostilbene, colistin, combination therapy

## Abstract

Carbapenem resistance among strains of the nosocomial pathogen *Klebsiella pneumoniae* is increasing worldwide, causing serious clinical infections and higher mortality rates. Polymyxins are some of the few “last resort” options for treatment of carbapenem-resistant *Enterobacteriaceae*, including *K. pneumoniae*, however, the emergence of plasmid-mediated colistin resistance gene *mcr-1* has largely rendered polymyxin-class antibiotics ineffective in a clinical setting. We previously identified a natural compound, pterostilbene, which has a synergistic effect in combination with polymyxins. Here, we aimed to determine whether pterostilbene application can restore the bactericidal activity of polymyxins against *mcr-1-*positive *K. pneumoniae*. Checkerboard MIC studies confirmed that pterostilbene reduces the MIC of colistin against *mcr-1-*positive clinical *K. pneumoniae* isolates, with the bacteria going from resistant to sensitive, and also demonstrated a synergistic effect with colistin (FIC index = 0.11 ± 0.04 or 0.28 ± 0.00). Time-killing assays showed that individually, both pterostilbene and colistin failed to eradicate *K. pneumoniae* strains, while in combination, the two drugs effectively eliminated *K. pneumoniae* ZJ02 and *K. pneumoniae* ZJ05 by 1–3 h post-inoculation. The combined disk test also showed increases in the zones of inhibition only for *mcr-1-*positive *Escherichia coli* and *K. pneumoniae* isolates. A mouse infection model demonstrated that the survival rate of mice at 7 days post-intraperitoneal injection with a lethal dose of *K. pneumoniae* ZJ05 was significantly promoted from 0 to 67% following combination therapy. This is the first time a MCR-1 inhibitor has successfully been used in combination with colistin against human clinical MCR-1 producing *K. pneumoniae* ZJ05 isolate.

## Introduction

The relentless increase in carbapenem-resistant *Enterobacteriaceae* (CRE) strains is now recognized as one of the most serious global threats to public health (Morrill et al., 2015). Carbapenem-resistant *K. pneumoniae* strains are especially worrying as they have higher morbidity and mortality rates, and treatment of these bacterial infections is frequently challenging because of the limited therapeutic options (Olaitan et al., 2014; Quan et al., 2017). *K. pneumoniae* is a common cause of pulmunary and bloodstream health care related infections and normally resides in the lower gastrointestinal tract, where it can acquire high-level antibiotic resistance (Hrabák et al., 2011). This eventuality forced a re-evaluation of the use of one of the earliest classes of antibiotics, polymyxins, for treatment of serious infections caused by carbapenem and multidrug resistant *K. pneumoniae* isolates often blaKPC or blaNDM- positive (Quan et al., 2017). In human clinical chemotherapy, polymyxin B and polymyxin E are usually used in combination, mainly because the dose escalation that is required to achieve sufficiently high concentrations under the currently recommended dosing protocols, risks the rapid onset of nephrotoxicity and neuromuscular blockade (Pogue et al., 2011).

Prior to the detection of the plasmid-mediated colistin resistance gene *mcr-1*, almost all studies of polymyxin resistance focused on the *pmrAB* and *phoPQ* two-component regulatory systems, inactivation of *mgrB*, or the lack of lipopolysaccharide (Halaby et al., 2013). *mcr-1* encodes a phosphoethanolamine transferase that alters the charge on lipid A from electronegative to electropositive, thereby inhibiting the binding of polymyxins to target bacteria. *mcr-1* determinant amongst CRE has almost eliminated their clinical susceptibility to polymyxin (Liu et al., 2016, 2017; Kieffer et al., 2017). Importantly, as *mcr-1* is plasmid-mediated, resistance to polymyxins is no longer only associated with the chromosome, but can also be acquired by horizontal transmission (Giamarellou, 2016).

The loss of these last-line-of-defense antibiotics made necessary of the development of novel and effective strategies to deal with the serious challenges posed by MCR-1 expression, with the investment of large amounts of manpower and resources. It would also be useful to restore the efficacy of polymyxin to treat severe clinical bacterial infections caused by CRE (Bulman et al., 2017). Previously, we showed that a natural compound used in traditional Chinese medicine, pterostilbene, which has been extensively studied for its potent anti-cancer, anti-inflammatory, and anti-oxidant activities (Roupe et al., 2006), has a synergistic effect with polymyxin B against *E. coli* both *in vitro* and *in vivo* (Zhou et al., 2018). Because of its methoxyl substitution-induced hyperlipophilicity, pterostilbene may have higher bioactivity than resveratrol, making it potentially advantageous as a therapeutic agent (Cichocki et al., 2008; Kapetanovic et al., 2011). Here, we further characterized the efficacy of pterostilbene administrated together with polymyxins, and showed that it can help restore the bactericidal activity of polymyxins against *mcr-1-*positive *K. pneumoniae*.

## Materials and methods

### Bacterial strains and chemicals

Human clinical MCR-1 producing isolates *K. pneumoniae* ZJ02, *K. pneumoniae* ZJ05 and *E. coli* ZJ40 were collected in our previous study (Wang et al., 2017). And the *mcr-1* gene was chromosomally located in *E. coli* ZJ40. *K. pneumoniae* E8.31, *K.pneumoniae* 13b5 and *K.pneumoniae* L18 were collected from food animals. We also used *E. coli* strain DH5α (pUC19-*mcr-1*) (Zhou et al., 2018), which carries a *mcr-1* gene originating from *K. pneumoniae* ZJ05. Polymyxin-resistant *mcr-1*-negative *K. pneumoniae* isolate 16ZJJ9-19BC was obtained from a chicken cloacae sample collected in Zhejiang, China. *E. coli* ATCC25922, *K. pneumoniae* ATCC700603 and *K. pneumoniae* K7 were used as quality control strains. Pterostilbene (≥97% HPLC-pure) was purchased from Sigma-Aldrich (St. Louis, MO, USA). Colistin sulfate, polymyxin B sulfate, penicillin, imipenem, gentamicin sulfate, and chloramphenicol were purchased from the National Institute for the Control of Pharmaceutical and Biological Products (Beijing, China). Cephalothin sodium, streptomycin sulfate, kanamycin sulfate, erythromycin, and acheomycin were purchased from Dalian Meilun Biotechnology Co. (Dalian, China). Stock solutions of pterostilbene were prepared in dimethyl sulfoxide (Sigma-Aldrich).

### MIC determination and growth curves

The MIC assays were used to identify synergies between pterostilbene and colistin against polymyxin-resistant strains (positive for *mcr-1*), polymyxin-resistant strains (negative for *mcr-1*), and polymyxin-sensitive strains (negative for *mcr-1*), and were carried out using the broth microdilution method following the guidelines of the Clinical and Laboratory Standards Institute (Wiegand et al., 2008; Espinel-Ingroff et al., 2016). The remaining nine antibiotics were also tested in combination with pterostilbene. The efficacies of the combinations were evaluated by calculating the fractional inhibitory concentration (FIC) index values (Ma et al., 2016). A growth curve assay was also performed to evaluate the effect of pterostilbene on the growth of the tested strains (Li et al., 2011). Briefly, *K. pneumoniae* ZJ02 and *K. pneumoniae* ZJ05 were cultured in Luria-Bertani (LB) medium at 37°C with shaking at 200 rpm to obtain an OD_600_ value of 0.3. Aliquots (250 mL) of the culture were then transferred into six 50-mL Erlenmeyer flasks, and pterostilbene (or the dimethyl sulfoxide control) was added to the cultures at 0, 16, 32, 64, and 128 μg/mL, respectively. The bacteria were cultured at 37°C with shaking, and bacterial growth was estimated by measuring the OD_600_ every 30 min.

### Time-killing assays

The potential bactericidal effect of pterostilbene in combination with colistin was evaluated by time-killing assays (Petersen et al., 2006). Mid-logarithmic-phase bacterial cells were diluted to 5 × 10^5^ CFU/mL in LB broth supplemented with colistin (4 μg/mL), pterostilbene (16 μg/mL), colistin (4 μg/mL) in combination with pterostilbene (16 μg/mL), or DMSO (normal control). Cultures were incubated at 37°C with shaking and samples were removed at 0, 1, 3, 5, and 7 h post-inoculation for bacterial counts. Serial 10-fold dilutions of the samples were spread onto LB agar plates without antibiotics. Bacterial colonies were counted following incubation at 37°C for 24 h.

### Combined disk test

The combined disk test (CDT) was carried out as described previously (Pournaras et al., 2013; Watts, 2013). Based on the results of the growth curve assay and checkerboard MIC studies, we selected pterostilbene concentrations of 0, 8, and 32 μg/mL, none of which resulted in an inhibitory effect against any of the screened strains. Colistin 10 μg disks (Oxoid Ltd., Basingstoke, United Kingdom) were first placed on Mueller-Hinton-Broth (MHB) agar plates inoculated with bacterial suspension at an OD_600_ = 0.1. Ten-microliter aliquots of the different concentrations of pterostilbene solution were then directly added to the disks. The diameters of the inhibition zones around the colistin disks (with and without pterostilbene) were measured and compared following incubation for 18–24 h at 37°C.

### *In vivo* infection model for *K. pneumoniae* ZJ05

A mouse model of endonasal pulmonary infection was used to determine the synergistic effect of pterostilbene in combination with colistin *in vivo*. Eight-week-old female C57BL/6J mice weighing 20 ± 2 g were obtained from the Experimental Animal Centre of Jilin University (Changchun, China). Animal experiments were approved by and conducted in accordance with the guidelines of the Animal Care and Use Committee of Jilin University. Five mice were housed per cage in a pathogen-free environment maintained at 24 ± 2°C and 50% ± 10% relative humidity and subjected to a 12 h light/12 h dark cycle. All mice were rested for 5 days prior to use to allow acclimatization.

Pneumonia was induced in the mice as described previously (Bowers et al., 2015; Zhou et al., 2017). *K. pneumoniae* ZJ05 was grown to mid-logarithmic phase (OD_600_ = 0.5) in LB medium at 37°C and then centrifuged at 5,000 × g for 5 min at 4°C. After washing three times with PBS, the bacteria were resuspended in PBS. The mice were divided randomly into five groups (solvent control for each treatment, pterostilbene alone, colistin alone, and pterostilbene in combination with colistin). Each experimental group contained 18 mice. For the survival experiments, the mice were lightly anesthetized by inhalation of isoflurane and then inoculated in the left nare with 20 μL of suspension containing 1 × 10^8^ CFU of the prepared *K. pneumoniae* ZJ05 cells. The infected mice were subcutaneously administered colistin (8 mg/kg), pterostilbene (50 mg/kg), a combination of pterostilbene (50 mg/kg) and colistin (8 mg/kg), or solvent on the same schedule at 2 h post-infection and then at 8-h intervals. Mice were monitored until day 7 post-infection.

For histopathological analysis of lung infection and calculation of the wet/dry weight ratio, mice were inoculated with 5 × 10^7^ CFU of prepared *K. pneumoniae* ZJ05 cells. The mice were killed with anesthesia followed by cervical dislocation at 48 h post-infection. Homogenates of lung tissue, which was collected from euthanized mice, were prepared in 1 ml of sterile PBS and used to calculate bacterial colony counts following serial dilution and smearing on LB agar plates. For histopathological analysis, the lungs were placed in 10% (v/v) formalin, followed by staining with hematoxylin and eosin and examination by light microscopy. The lungs were isolated to measure the wet weight, while the dry weight was measured after drying for 72 h at 70°C. The wet/dry weight ratio of the lung was then calculated.

### Statistical analysis

The IBM Statistical Program for Social Sciences (SPSS) version 19.0 (IBM Corp. Armonk, NY, USA) was used to analyze experimental data, and data are presented as the mean ± standard deviation. An independent Student's *t*-test was used to determine significant differences, and differences were considered statistically significant when *P-*values were less than 0.05.

## Results

### Pterostilbene showed a synergistic effect in combination with polymyxin against *mcr-1-* positive bacteria

**Figure 1:**
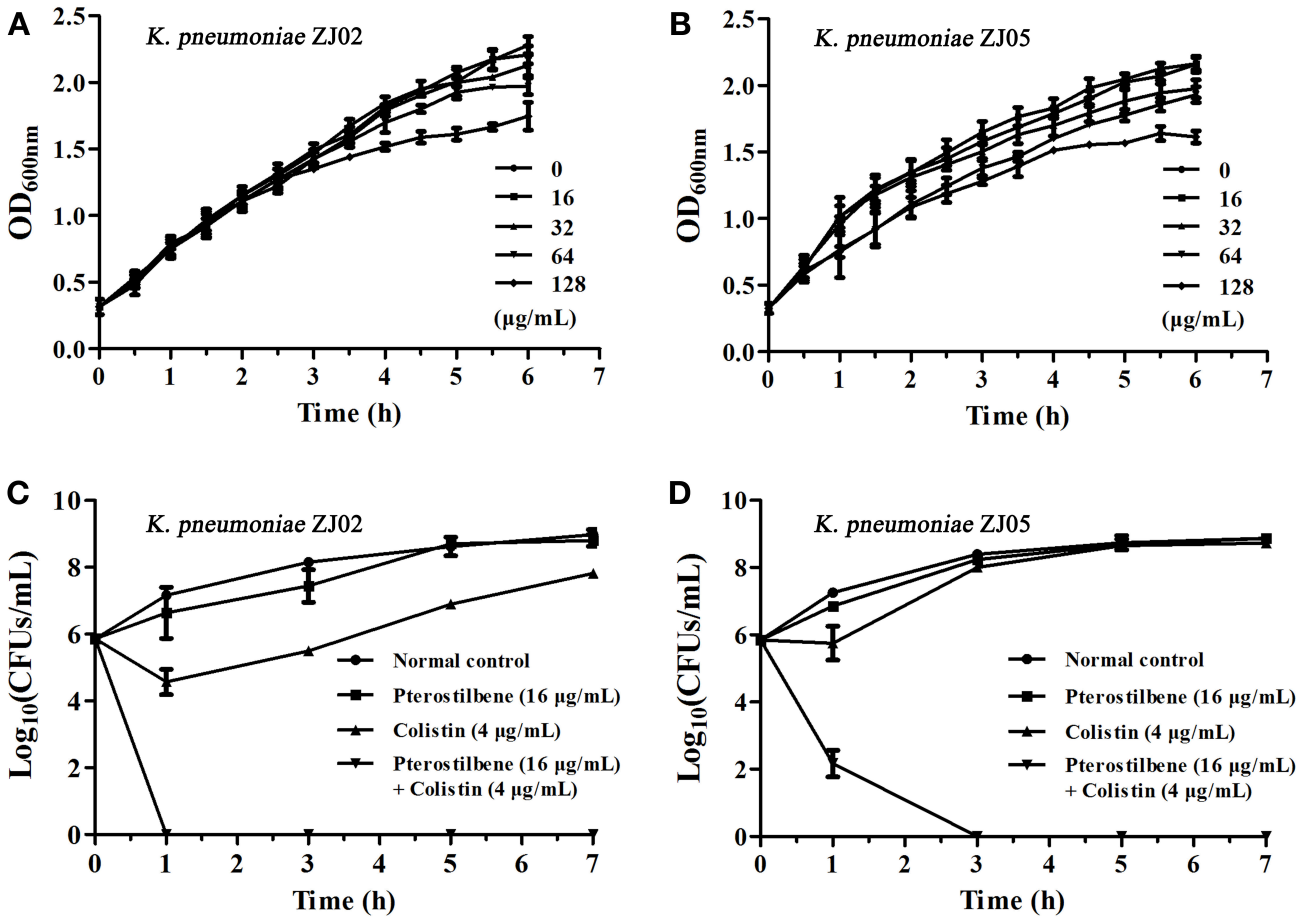
Pterostilbene in combination with colistin restores the *in vitro* sensitivity of *K. pneumoniae* to polymyxins. **(A,B)** Growth curves for *K. pneumoniae* ZJ02 **(A)** and *K. pneumoniae* ZJ05 **(B)** cultured in the presence of various concentrations (0–128 μg/mL) of pterostilbene. Values represent the averages of three independent experiments. **(C,D)** Time-killing curves for colistin, pterostilbene, colistin + pterostilbene, and control treatment (medium only) against *K. pneumoniae* ZJ02 **(C)** and *K. pneumoniae* ZJ05 **(D)**. Values represent the averages of three independent experiments.

We previously showed that pterostilbene (trans-3,5-dimethoxy-4′-hydroxystilbene) had a synergistic effect with polymyxin B and colistin against polymyxin-resistant *E. coli* strains (positive for MCR-1) (Zhou et al., 2018). In view of the clinical significance of *K. pneumoniae*, and to determine the synergistic effect of pterostilbene in combination with polymyxin alone, *mcr-1*-positive *K. pneumoniae* isolates ZJ02, ZJ05, E8.31, 13B5, and L18 were examined in this study. Our results confirmed the synergistic effect of pterostilbene only in combination with colistin against both *mcr-1*-positive clinical *K. pneumoniae* isolates (FIC = 0.11 ± 0.04–0.28 ± 0.00, respectively, in the presence of 16 μg/mL of pterostilbene) using the broth microdilution checkerboard method. No synergy was observed with any of the other nine tested antibiotics against either the *mcr-1*-positive or polymyxin-sensitive isolates. However, the synergistic effect of pterostilbene and polymyxin against *mcr-1*-negative polymyxin-resistant *K. pneumoniae* strain 16ZJJ9-19BC differed from that observed using *mcr-1-*positive isolates (Table 1). The growth curve showed that none of the concentrations of pterostilbene (0–128 μg/mL) affected the growth of *mcr-1*-positive *K. pneumoniae* isolates ZJ02 and ZJ05 (Figures 1A,B).

**Table 1:** MIC values for the different antibiotics used alone or in combination with pterostilbene against each of the tested bacterial isolates.

**Species**	**Source and *mcr-1* confirmation**	**Antibiotics**	**MIC (**μ**g/mL)**		**FIC Index**
			**Alone**	**Combination**	
*K. pneumoniae* ZJ02	*mcr-1*-carrying *K. pneumoniae* from clinical infections in Zhejiang	Colistin	16.00 ± 0.00	1.33 ± 0.58	**0.11** ± **0.04**
		Cefalotin sodium	1024.00 ± 0.00	1024.00 ± 0.00	1.03 ± 0.00
		Penicillin	1024.00 ± 0.00	1024.00 ± 0.00	1.03 ± 0.00
		Imipenem	2.67 ± 0.00	2.67 ± 0.00	1.03 ± 0.00
		Streptomycin	512.00 ± 0.00	512.00 ± 0.00	1.03 ± 0.00
		Kanamycin	1024.00 ± 0.00	1024.00 ± 0.00	1.03 ± 0.00
		Gentamycin	512.00 ± 0.00	512.00 ± 0.00	1.03 ± 0.00
		Chloramphenicol	512.00 ± 0.00	512.00 ± 0.00	1.03 ± 0.00
		Erythromycin	256.00 ± 0.00	256.00 ± 0.00	1.03 ± 0.00
		Acheomycin	213.33 ± 73.90	213.33 ± 73.90	1.20 ± 0.76
*K. pneumoniae* ZJ05	*mcr-1*-carrying *K. pneumoniae* from clinical infections in Zhejiang	Colistin	26.67 ± 9.24	2.67 ± 1.15	**0.14** ± **0.04**
		Cefalotin sodium	1024.00 ± 0.00	1024.00 ± 0.00	1.03 ± 0.00
		Penicillin	1024.00 ± 0.00	1024.00 ± 0.00	1.03 ± 0.00
		Imipenem	1.00 ± 0.00	1.00 ± 0.00	1.03 ± 0.00
		Streptomycin	21.33 ± 9.24	21.33 ± 9.24	1.03 ± 0.00
		Kanamycin	26.67 ± 9.24	26.67 ± 9.24	1.03 ± 0.00
		Gentamycin	3.33.00 ± 1.15	2.67 ± 1.15	0.86 ± 0.29
		Chloramphenicol	5.33 ± 2.31	5.33 ± 2.31	1.03 ± 0.00
		Erythromycin	128.00 ± 0.00	128.00 ± 0.00	1.03 ± 0.00
		Acheomycin	170.67 ± 73.90	170.67 ± 73.90	1.03 ± 0.00
*E. coli* DH5α (pUC19-*mcr-1*)	Laboratory strain (carried a *mcr-1* gene that originated from *K. pneumoniae* ZJ05)	Colistin	13.33 ± 4.62	2.00 ± 0.00	**0.20** ± **0.07**
		Cefalotin sodium	256.00 ± 0.00	256.00 ± 0.00	1.03 ± 0.00
		Penicillin	512.00 ± 0.00	512.00 ± 0.00	1.03 ± 0.00
		Imipenem	0.25 ± 0.00	0.25 ± 0.00	1.03 ± 0.00
		Streptomycin	2.00 ± 0.00	2.00 ± 0.00	1.03 ± 0.00
		Kanamycin	2.67 ± 1.15	2.67 ± 1.15	1.03 ± 0.00
		Gentamycin	1.67 ± 0.58	1.67 ± 0.58	1.03 ± 0.00
		Chloramphenicol	4.00 ± 0.00	4.00 ± 0.00	1.03 ± 0.00
		Erythromycin	16.00 ± 0.00	16.00 ± 0.00	1.03 ± 0.00
		Acheomycin	1.00 ± 0.00	1.00 ± 0.00	1.03 ± 0.00
*E. coli* DH5α (pUC19)	Laboratory strain (Polymyxin-sensitive *mcr-1*-negative)	Colistin	0.83 ± 0.29	0.67 ± 0.89	0.86 ± 0.29
		Cefalotin sodium	256.00 ± 0.00	256.00 ± 0.00	1.03 ± 0.00
		Penicillin	512.00 ± 0.00	512.00 ± 0.00	1.03 ± 0.00
		Imipenem	0.25 ± 0.00	0.25 ± 0.00	1.03 ± 0.00
		Streptomycin	2.00 ± 0.00	2.00 ± 0.00	1.03 ± 0.00
		Kanamycin	2.00 ± 0.00	2.00 ± 0.00	1.03 ± 0.00
		Gentamycin	1.67 ± 0.58	1.67 ± 0.58	1.03 ± 0.00
		Chloramphenicol	4.00 ± 0.00	4.00 ± 0.00	1.03 ± 0.00
		Erythromycin	8.00 ± 0.00	8.00 ± 0.00	1.03 ± 0.00
		Acheomycin	1.00 ± 0.00	1.00 ± 0.00	1.03 ± 0.00
*E. coli* ZJ40	*mcr-1*-carrying *K. pneumoniae* from clinical infection in Zhejiang (*mcr-1* located in chromosome)	Colistin	85.33 ± 36.95	3.33 ± 1.15	**0.15** ± **0.05**
		Polymyxin B	53.33 ± 18.48	2.67 ± 1.15	**0.18** ± **0.02**
*K. pneumoniae*-E8.31	Polymyxin-resistant *mcr-1*-positive *K. pneumoniae* from chicken cloacae in Shandong	Colistin	21.33 ± 9.24	3.33 ± 0.00	**0.20** ± **0.07**
		Polymyxin B	16.00 ± 0.00	2.67 ± 1.15	**0.20** ± **0.07**
*K. pneumoniae*-L18	Polymyxin-resistant *mcr-1*- positive *K. pneumoniae* from chicken cloacae	Colistin	13.33 ± 4.62	3.33 ± 1.15	**0.28** ± **0.00**
		Polymyxin B	16.00 ± 0.00	2.67 ± 1.15	**0.20** ± **0.07**
*K. pneumoniae*-13b5	Polymyxin-resistant *mcr-1*- positive *K. pneumoniae* from chicken cloacae in Shanghai	Colistin	32.00 ± 0.00	3.33 ± 1.15	**0.14** ± **0.04**
		Polymyxin B	26.67 ± 9.24	2.67 ± 1.15	**0.14** ± **0.04**
*K. pneumoniae*−16ZJJ9-19BC	Polymyxin-resistant *mcr-1*-negative *K. pneumoniae* from chicken cloacae in Zhejiang	Colistin	32.00 ± 0.00	10.67 ± 4.62	0.36 ± 0.14
		Polymyxin B	26.67 ± 9.24	10.67 ± 4.62	0.45 ± 0.14
*K. pneumoniae* K7	*mcr-1*-negative *K. pneumoniae* from clinical infection in Jilin	Colistin	1.33 ± 0.58	1.33 ± 0.58	1.03 ± 0.00
		Polymyxin B	2.00 ± 0.00	2.00 ± 0.00	1.03 ± 0.00
*K. pneumoniae* ATCC700603	Laboratory strain	Colistin	0.67 ± 0.29	0.83 ± 0.29	1.36 ± 0.58
		Polymyxin B	1.00 ± 0.00	1.00 ± 0.00	1.03 ± 0.00

*All MICs were determined in triplicate. According to the best synergistic effect, pterostilbene was used at a concentration of 16 μg/mL for K. pneumoniae and 32 μg/mL for E. coli, except E. coli ZJ40 (4 μg/mL). The FIC values of all mcr-1-positive isolates were indicated in bold*.

**Figure 2:**
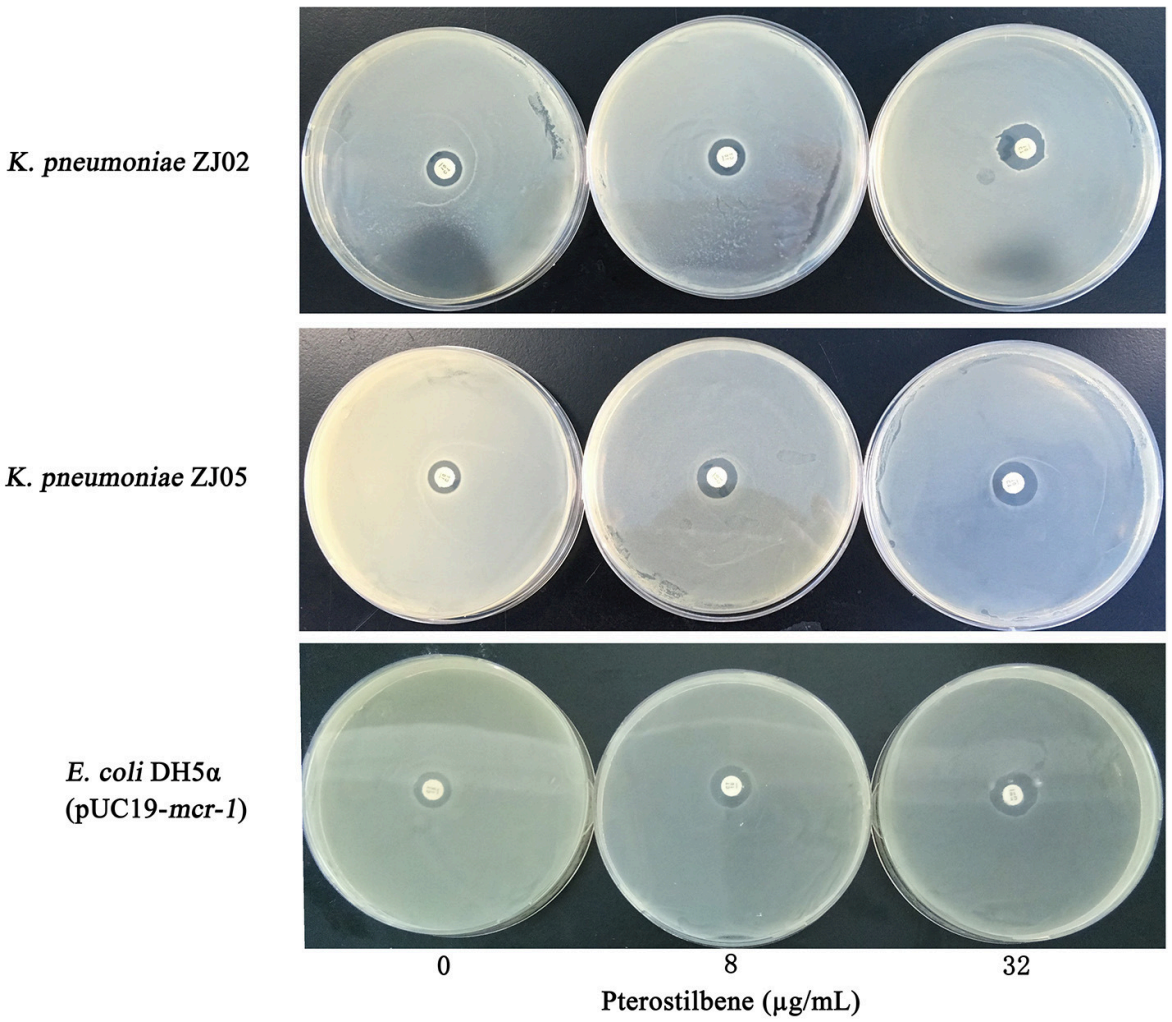
Zones of inhibition surrounding colistin disks supplemented with 0, 8, or 32 μg/mL of pterostilbene on lawns of *K. pneumoniae* ZJ02, *K. pneumoniae* ZJ05, and *E. coli* DH5α (pUC19*-mcr-1*) on MHB agar plates.

The combination of pterostilbene and colistin resulted in the lowest FIC index value, and thus was examined further via time-killing assays. The time-killing assays were performed using 16 μg/mL of pterostilbene and 4 μg/mL of colistin against *K. pneumoniae* grown in LB broth. When used alone, pterostilbene and colistin had little effect on bacterial growth. In contrast, the combination of pterostilbene and colistin resulted in the elimination of *K. pneumoniae* ZJ02 and *K. pneumoniae* ZJ05 at 1 h and 3 h post-administration, respectively (Figures 1C,D). Based on the results of the growth curve, pterostilbene concentrations of 0, 8, and 32 μg/mL were chosen for CDT assays. The results showed increases in the zones of inhibition only for *mcr-1*-positive *E. coli* and *K. pneumoniae* isolates (2.67 ± 0.58 mm, 4.33 ± 0.29 mm, and 4.67 ± 0.29 mm) using disks containing 10 μg of colistin plus 32 μg/mL of pterostilbene in comparison with the inhibition zones of disks containing 10 μg of colistin alone (Table 2 and Figure 2), and *mcr-1*-negative *E. coli* ATCC 25922 had little increases in the zones of inhibition with different concentrations of pterostilbene. We also confirmed that pterostilbene in combination with colistin increased the size of the inhibition zones in a dose-dependent manner.

**Table 2:** Combined disk test for colistin in combination with pterostilbene for each of the tested bacterial isolates.

**Specices**	**Inhibition zone diameter (mm)**					
	**Assay**	**Colistin (10 μg)**	**Colistin (10 μg) + Pterostilbene (8 μg/mL)**	**Increase**	**Colistin (10 μg) + Pterostilbene (32 μg/mL)**	**Increase**
*K. pneumoniae* ZJ05	Assay 1	8.5	11.5	**3**	13.0	**4.5**
	Assay 2	9.5	11.5	**2.0**	14.0	**4.5**
	Assay 3	9.0	11.0	**2.0**	14.0	**5.0**
	Mean	*9.0 ± 0.50*	11.33 ± 0.29^**^	***2.33** ± **0.58***	13.67 ± 0.58^**^	***4.67** ± **0.29***
*K. pneumoniae* ZJ02	Assay 1	9.5	11.0	**1.5**	13.5	**4**
	Assay 2	9.0	10.5	**1.5**	13.5	**4.5**
	Assay 3	9.0	11.0	**2**	13.5	**4.5**
	Mean	*9.17 ± 0.29*	10.83 ± 0.29^**^	***1.67** ± **0.29***	13.50 ± 0.00^**^	***4.33** ± **0.29***
*E. coli* DH5α (pUC19-*mcr-1*)	Assay 1	10.5	12.0	**1.5**	13.5	**3.0**
	Assay 2	10.0	11.0	**1.0**	13.0	**3.0**
	Assay 3	11.0	11.5	**0.5**	13.0	**2.0**
	Mean	*10.0 ± 0.50*	*11.50 ± 0.50*	***1.00** ± **0.50***	13.17 ± 0.29^**^	***2.67** ± **0.58***
*E. coli* ATCC 25922	Assay 1	13.0	13.5	**0.5**	13.5	**0.5**
	Assay 2	12.5	13.5	**1.0**	12.0	**-0.5**
	Assay 3	13.0	13.0	**0.0**	13.5	**0.5**
	Mean	*12.83 ± 0.89*	*13.33 ± 0.29*	***0.50** ± **0.50***	*13.00 ± 0.87*	***0.17** ± **0.58***

*The combined disk test method was performed in triplicate. Three 10-μg colistin disks with pterostilbene (0, 8, and 32 μg/mL) were used*.

** *P < 0.01 compared with the colistin 10-μg disk alone based on two-tailed Student's t-tests. The mean inhibition zone diameter of all isolates were indicated in italics, and the increased values were indicated in bold*.

### Combination therapy had a synergistic effect *in vivo* in comparison with monotherapy or the control

**Figure 3:**
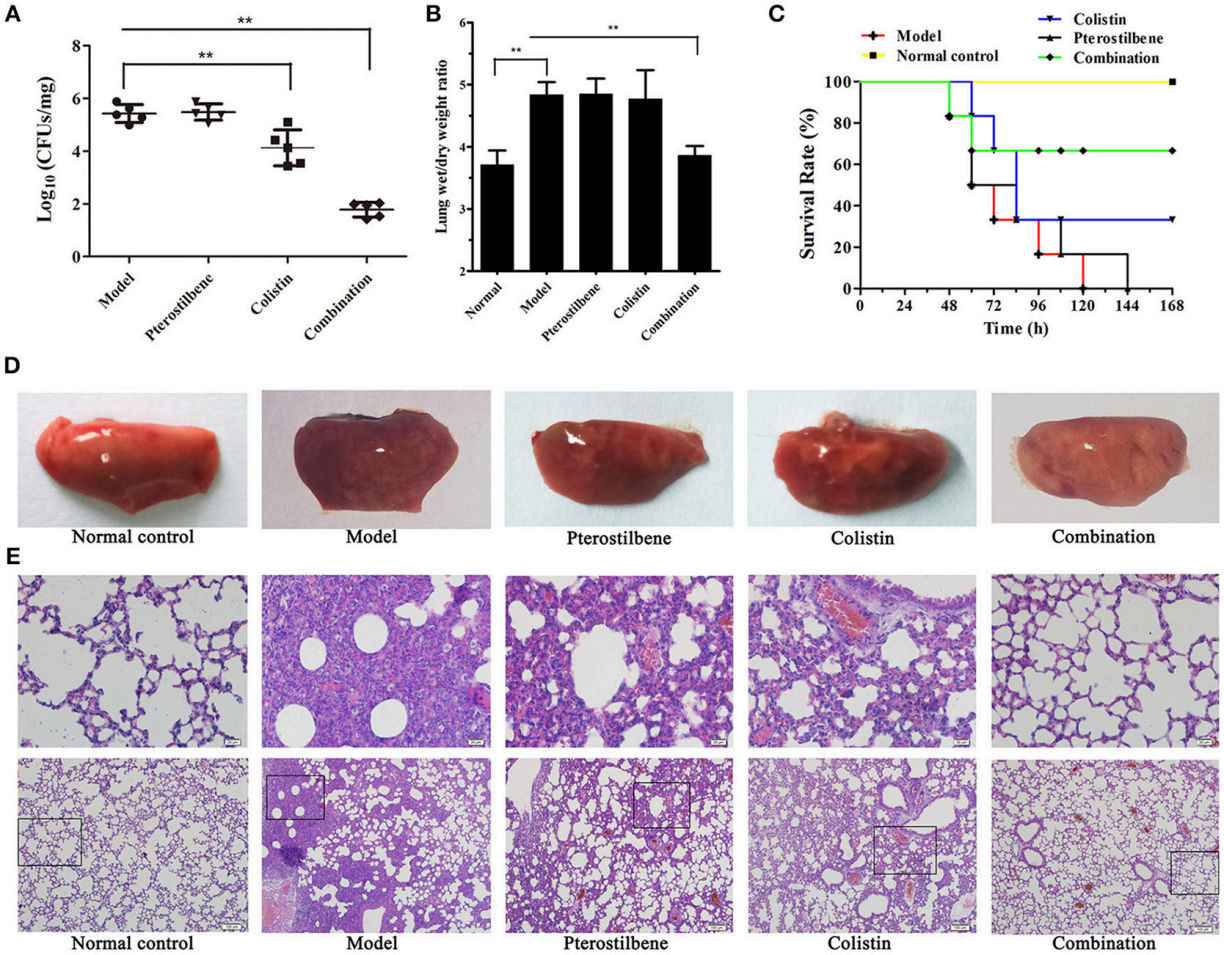
Effects of pterostilbene and colistin combination therapy *in vivo*. Mice were infected with *K. pneumoniae* ZJ05 and then treated with pterostilbene, colistin, pterostilbene combined with colistin (combination), or control solvent treatment (model). Uninfected mice were used as a healthy control (normal control). At 48 h post-inoculation, mice were euthanized and the bacterial burden **(A)** and wet/dry weight ratio **(B)** of lungs were calculated. **P < 0.01. **(C)** Survival curve of mice infected with *K. pneumoniae* ZJ05 and monitored for 7 days post-infection. The data represent the means and standard deviations from three separate experiments (18 mice per group). Gross pathological changes **(D)** and histopathology **(E)** of the lung tissue of mice from the first experiment were also assessed.

Based on the above results, we attempted to determine whether the synergistic effects could be replicated *in vivo* in a mouse model of pneumonia induced by *K. pneumoniae*. Mice were intranasally inoculated with *K. pneumoniae* ZJ05 and then treated with colistin (8 mg/kg), pterostilbene (50 mg/kg), pterostilbene (50 mg/kg) in combination with colistin (8 mg/kg), or PBS as a control at 2 h post-infection, and bacterial burden was assessed at 24 h post-infection. The combination of colistin and pterostilbene resulted in a significant reduction of the bacterial load in the lung compared with the monotherapy treatments (*P* < 0.01; Figure 3A), although the colistin-treated group also showed a significant decrease in CFU compared with the control group (*P* < 0.01).

We assessed the degree of pulmonary edema via the wet/dry weight ratio of the left lung. The results showed that mice treated with the combination therapy had a significant decrease in wet/dry weight ratio compared with the other groups (Figure 3B). Histopathological analysis of lung tissue was also performed to evaluate the treatment efficacy of pterostilbene in combination with colistin against pulmonary injury. Gross macroscopic inspection revealed that the lungs of infected mice that receiving either of the monotherapies or the control treatment were crimson and exhibited severe congestion and pulmonary edema. In contrast, the lung tissue of mice treated with combination therapy remained pink and fungous (Figure 3D). Examination of the pathologic manifestations (Figure 3E) revealed that the infected mice in either the untreated or monotherapy-treated groups exhibited severe tissue injury and inflammatory cell aggregation. In contrast, the tissue sections of the mice in the combination therapy group were similar to those of the normal mice.

The combination therapy was further tested using a mouse survival model. Following infection with 1 × 10^8^ CFU of *K. pneumoniae* ZJ05, the majority of mice treated with a single agent or the control succumbed to infection within 168 h. However, as shown in Figure 3C, 67% (12/18) of the mice treated with a combination of pterostilbene and colistin survived until the end of the experiment.

## Discussion

Because of the significant burden of *mcr-1*-positive *K. pneumoniae* in a clinical setting, we investigated whether pterostilbene in combination with colistin could be used as a treatment for infections caused by colistin-resistant *K. pneumoniae*. Pterostilbene (trans-3,5-dimethoxy-4′-hydroxystilbene) is a naturally occurring phytoalexin found in several plant species. It has more favorable pharmacological properties than fellow phytoalexin resveratrol, including greater oral absorption efficiency, potential for greater cellular uptake, and a longer half-life. Moreover, it exhibits antibacterial activity against drug-resistant *Staphylococcus aureus* strains without inducing unacceptably high levels of cytotoxicity (0.125 mM) in mammalian cells. For example, administration of pterostilbene (3,000 mg/kg, daily, p.o.) for approximately 30 days did not result in remarkable local or systemic toxicity in mice. Another study showed that pretreatment of A/J mice with pterostilbene at doses of 50 and 250 mg/kg (i.p.) five times per week for 21 continuous weeks produced no signs of toxicity, such as changes in fur color, motor or behavioral abnormalities, or palpable masses (Chen et al., 2012). Pterostilbene is also generally safe for human consumption at doses of up to 250 mg per day, and is used as a dietary supplement to decrease the risk of coronary heart disease (Riche et al., 2013). Therefore, all studies confirm that pterostilbene has no measurable toxicity in animals or humans, regardless of the route of administration, and suggest that this natural compound is likely to be safe if applied in human clinical practice.

Although there is a significant synergistic effect of pterostilbene in combination with polymyxin, it is not enough to warrant the development of a therapeutic agent for clinical use. Therefore, it is necessary to study the molecular structure of pterostilbene, including modifications of the main chemical functional groups, which may be useful for reducing any potential side effects for clinical use. There are several limitations to the use of pterostilbene, including its low bioavailability and poor water solubility (Chen et al., 2012). However, compared with resveratrol, pterostilbene has a higher bioavailability and is processed more slowly (glucuronidated or sulfated) *in vivo*, which may increase the functionality of pterostilbene when applied in systemic infections (Chiou et al., 2011).

The mechanisms of resistance to polymyxins, including mutations in the PmrAB/PhoPQ two-component regulatory systems, loss of lipopolysaccharide, MgrB inactivation, and plasmid-mediated colistin resistance, all involve the modification of lipid A, resulting in a reduction of polymyxin affinity(Ah et al., 2014; Antonelli et al., 2017). A variety of polymyxin resistance mechanisms are present in *Enterobacteriaceae* species, with some strains containing two or more pathways (Baron et al., 2016; Poirel et al., 2017). Therefore, we need to further explore the mechanism of resistance in *mcr-1*-negative colistin-resistant *K. pneumoniae* isolates. In the current study, we used several standard methods to determine the synergy of pterostilbene, including disk diffusion assays carried out as described by the Clinical and Laboratory Standards Institute. This technique is still used for *in vivo* susceptibility testing in many countries despite the fact that polymyxins do not readily diffuse in agar, resulting in reduced reliability of the method for measuring MIC (Boyen et al., 2010; Albur et al., 2014; Esposito et al., 2017). Despite the limitations of this assay method, we observed significant differences in MCR-1-producing isolates *K. pneumoniae* ZJ02, *K. pneumoniae* ZJ05, and *E. coli* DH5α (pUC19-*mcr-1*) compared with *E. coli* ATCC25922.

In summary, this study shows that a combination of polymyxins and pterostilbene could be a viable alternative treatment option for combating *K. pneumoniae* strains harboring mobile polymyxin resistance gene *mcr-1*. In addition, this alternative strategy provides potential opportunities to abate pathogenicity and its consequences without placing selective pressure on the target bacterium (Song et al., 2017). Furthermore, by reducing the amount of polymyxins used in clinical therapy, this strategy may also decrease the possibility of mutations arising in LPS modification pathways in *K. pneumoniae*, as can occur following long-term use of polymyxins. Further studies, including elucidation of the mechanism of inhibition of MCR-1 by pterostilbene, are needed to optimize the effects of combination therapy.

## Conclusion

In this study, we identified a natural compound of a Traditional Chinese Medicine, pterostilbene, when used in combination with colistin, regain its bactericidal activity against the *mcr*-*1-*positive *K. pneumoniae*. The microdilution checkerboard method confirmed that the pterostilbene reduces the MIC of colistin in *mcr-1*-positive *K. pneumoniae* strains from resistance to sensitive. The time-killing assays showed that either pterostilbene or colistin failed to eradicate ZJ02 and ZJ05, but the combination eliminated ZJ02 and ZJ05 by 1–3 h post-inoculation. The mouse infection model demonstrated that the survival rate of mice following the infection with ZJ05 was significantly promoted from 0% in the group of the administrated as monotherapy to 67% in the group of combination therapy applied.

## Author contributions

XD, YW, and YZ: Study design. YZ, TW, and YG: Experimental studies. SL, YS, and ST: Data analysis, interpretation. YZ and JW: Statistical analysis. XD, YW, and YZ: Manuscript preparation.

### Conflict of interest statement

The authors declare that the research was conducted in the absence of any commercial or financial relationships that could be construed as a potential conflict of interest.
